# Chemically recycled commercial polyurethane (PUR) foam using 2-hydroxypropyl ricinoleate as a glycolysis reactant for flexibility-enhanced automotive applications†

**DOI:** 10.1039/d4ra04972a

**Published:** 2024-09-20

**Authors:** Vojtěch Jašek, Petr Montag, Přemysl Menčík, Radek Přikryl, Alena Kalendová, Silvestr Figalla

**Affiliations:** a Institute of Materials Chemistry, Faculty of Chemistry, Brno University of Technology 61200 Brno Czech Republic xcjasekv@vutbr.cz; b Tomas Bata University in Zlin, Faculty of Technology, Department of Polymer Engineering 76001 Zlín Czech Republic; c BASF Ltd Czech Republic

## Abstract

The automotive industry uses polyurethane (PUR) foam core in the vehicle headliner composite. The sector demands recycling suggestions to reduce its scrap and decrease the expenses. This work investigated the PUR depolymerization using synthesized 2-hydroxypropyl ricinoleate (2-HPR) from castor oil and incorporated the liquid recyclate (REC) into the original PUR foam. The synthesis of 2-HPR yielded 97.5%, and the following PUR depolymerization (*via* glycolysis) reached 87.2% yield. The synthesized products were verified by GPC, FTIR, ESI-MS, and ^1^H NMR cross-analysis. The laboratory experiments (565 mL) included rheological, structural, and reactivity investigations. Added 30% REC content decreased the apparent viscosity to 109 mPa s from standard 274 mPa s. The reactivity of the 30% REC system increased by 51.2% based on the cream time due to the high REC amine value. The block foam density of systems with 15% REC and above decreased by 14.8%. A system with 20% REC content was the most prospective for up-scale. The industrially significant up-scale (125 L) was performed successfully, and the tensile and flexural test specimens were sampled from the up-scaled foam. The tensile characteristic (tensile strength 107 ± 8 kPa and elongation 9.2 ± 0.7%) and flexural characteristic (flexural strength 156 ± 12 kPa and flexural strain at deformation limit 23.4 ± 0.6%) confirmed that the REC incorporation in the standard PUR foam improves the applicable significant mechanical properties and assures the manufacture improve.

## Abstract 2

Chemical recycling is a material and energy-saving concept that solves the landfilling and incinerating of used materials. This article presents the polyurethane chemical recycling route, including a bio-based solvent.

## Graphical abstract

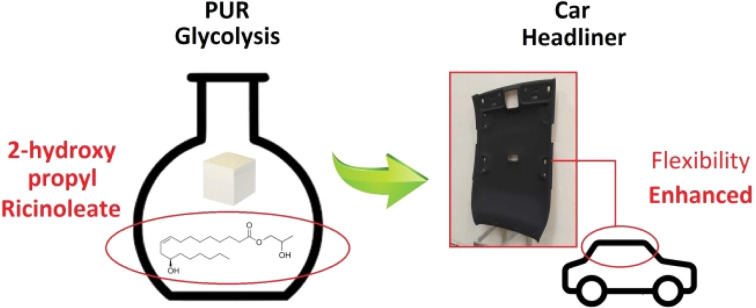

## 1 Introduction

Polyurethanes (PUR) are one of the most applied polymeric materials in the automotive industry. Since many PUR macromolecular types are distinguished, various components are fabricated and used mainly in the car interior, serving many purposes.^1–3^ Generally, polyurethanes are polymeric materials consisting of various alcohol structures (polyethers, polyesters) and certain types of isocyanates (toluene diisocyanate, methylene diphenyl diisocyanate, *etc.*), which are polymerized *via* addition reaction forming the polyurethane bonding, predominantly catalyzed by a basic catalyst (mainly amines).^4–7^ Carbon dioxide (CO_2_) is formed during the isocyanate reaction with water, blowing the mixture.^8^ Therefore, most polymerized polyurethanes are foam materials.^9^ Regarding the component's composition, many different kinds of PURs are manufactured.^4–7^ Flexible PUR foams contain an excess of long-chain polyol, which increases their flexibility due to the molecular mobility of the long terminal carbon backbones.^10,11^ Also, the excess of polyol in the structure increases the hydrophilic character of the material.^12^ These foams are usually used as a cushion material in the vehicle interior due to their material character. Various seats, armrests, and pillows mainly consist of flexible PURs.^13^ Rigid PURs incorporate the majority of the isocyanate content.^14^ Therefore, the molecular structure of rigid foams is more cross-linked, resulting in the materials' enhanced mechanical strength.^14,15^ These materials are mainly car thermal insulators (surrounding the motor or applied in the car trunk).^16,17^ Rigid and semi-rigid foams fulfill the vehicle's acoustic barrier role, particularly in the car interior surface decors. The formed foam cells can absorb the sound waves and reduce noise inside the car.^18–20^ Thermoplastic polyurethanes (TPU) don't occur as foam materials since their molecular structure is not formed like the flexible/rigid PUR foams.^21,22^ TPU is a thermoplastic material extruded and manufactured mechanically at higher temperatures.^23^ This type of PUR is often incorporated into a car interior as console car parts, various operation panels, or gear knobs.^24^

Chemical recycling of polymeric materials attracts much attention due to the European legislation for automotive setting particular targets regarding the recycled content in automotive products over the upcoming years.^25^ Many fundamental principles of PUR chemical recycling were described and investigated. The chemical reaction approaches such as hydrolysis,^26^ alcoholysis,^27^ acidolysis,^28^ or aminolysis of polyurethane materials were widely studied and reviewed.^29^ The particular type of PUR alcoholysis is called glycolysis.^30^ Glycolysis uses glycerol or glycol derivatives as nucleophilic substitution agents, which react with the formed urethane bonds and replace the initially bonded polyols in the structure.^31,32^ Numerous glycolysis investigations were published in the literature. The glycolysis of PURs using glycerol,^33^ ethylene glycol,^34^ diethylene glycol,^35^ or other short-chained polyols is the most studied approach. The glycerol and glycol derivatives containing fatty acids (glycerides) for glycolysis are often studied since they are bio-based (oils and fats), and they can be obtained as secondary materials or wastes (waste cosmetic oils, used cooking oils). These glycerides can serve as glycolysis reactive agents incorporated into the chemical recycling of PURs.^36,37^ Castor oil is a prospective glycolysis reactant since it contains the vacant hydroxyl group in its carbon backbone (see Fig. 2). The castor oil's transesterification results in the formation of glycerides, which were previously used for laboratory-scale flexible PUR production.^38^

PUR chemical recycling is a promising way to reduce incinerated and landfilled polyurethane waste. The producer can benefit from the decrease in virgin polyol content, and the chemically recycled material can be applied to manufacturing. According to the literature, only 29.7% of PUR is recycled. Then, 39.5% of all PUR waste is incinerated, and 30.8% is landfilled. The total produced PUR in the EU is 26 million metric tons (2022).^39^ Although glycolysis was numerously studied, 2-hydroxypropyl ricinoleate was not yet used for PUR chemical recycling. The structural alternative, 2-hydroxyethyl ricinoleate, was studied in different applications, such as drug delivery.^40^ The potential products' flexibility enhancement is the main advantage of 2-hydroxypropyl ricinoleate usage for PUR chemical recycling. Previously mentioned nucleophiles (glycerides, acids, amines) form highly rigid thermosets suitable for specific applications. On the other hand, the targeted vehicle headliners require a particular level of flexibility level for optimal product manufacture. The 2-hydroxypropyl incorporation into the PUR structure may improve this requirement.

**Fig. 1:**
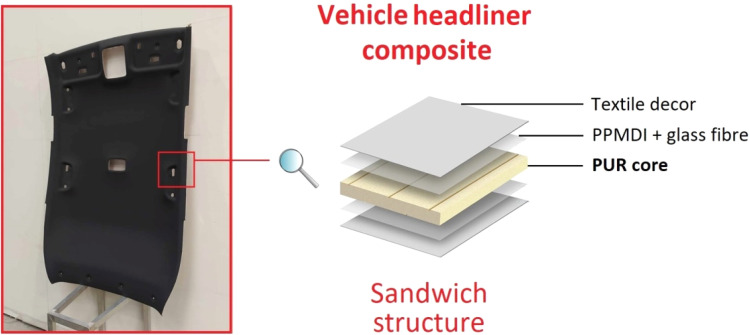
The vehicle headliner composition including the studied polyurethane (PUR) foam.

This article focuses on synthesizing 2-hydroxypropyl ricinoleate (2-HPR) as propylene glycol and castor oil transesterification product further used as a glycolysis reactant. The structural characterization of 2-HPR was confirmed *via*^1^H NMR, GPC, and FTIR. The commercial semi-rigid PUR foam – Elastoflex 3943/134/LD (produced by BASF Ltd) was selected for chemical recycling *via* glycolysis using microwave irradiation. The obtained liquid recyclate (REC) was incorporated into the original virgin polyol mixture, and the reactivity influence of the present REC was investigated *via* cup tests. The industrially most perspective polyol mixture containing PUR recyclate was selected based on the cup test results, and an up-scaled PUR foam 125 L block was formed. The foam block represents the industrially manufactured product used in the vehicle headliners (see Fig. 1). The structural differences between virgin and REC-containing foam were investigated *via* FTIR. The tension and flexural normalized tests investigated the REC-containing produced foam's application capability. A comparison of the mechanical properties of virgin and REC-containing foam was provided. This article discusses the advantages and limitations of the REC-containing foam application potential based on comparisons of the obtained reactivity, structure, and mechanical properties.

**Fig. 2:**
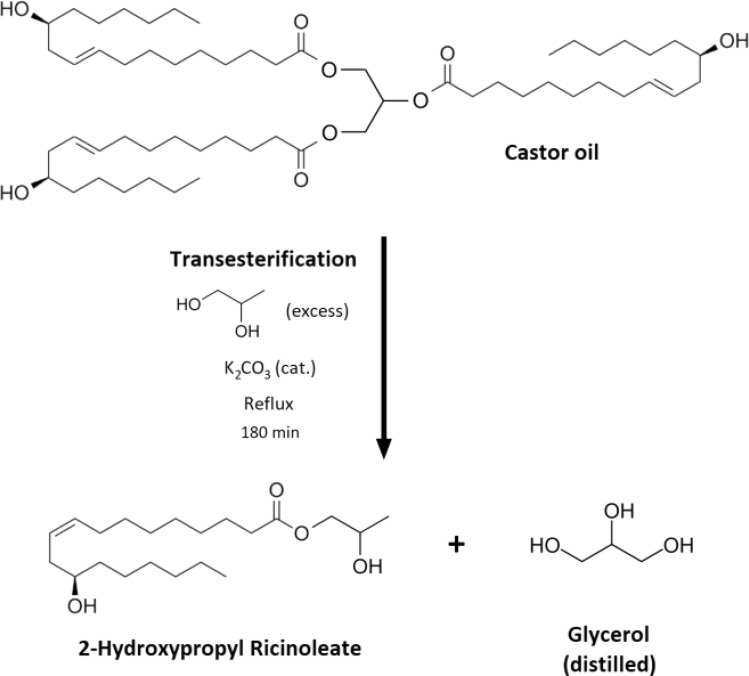
The reaction scheme of the castor oil transesterification resulting in the formation of 2-hydroxypropyl ricinoleate (2-HPR).

## 2 Experimental section

### 2.1 Materials

Castor oil (95.5% ricinoleic acid) was used for the transesterification, and propylene glycol (C_3_H_8_O_2_, 99%) was obtained from Fichema Ltd, Czech Republic. The catalyst for the glycolysis and transesterification – potassium carbonate (K_2_CO_3_, p.a.) was purchased from PENTA Ltd Czech Republic. Chemically recycled polyurethane foam scrap (Elastoflex 3943/134/LD), the commercial virgin polyol (E 3943/134/LD) ether-based system possessing the hydroxyl value of 288.0 mg KOH per g, and the commercial isocyanate systems (poly(methylene diphenyl diisocyanate)) (PMDI) system ISO 123/6) with average functionality of 2.7 were kindly received from the original producer – BASF Ltd, Germany. Chloroform (CHCl_3_ (stab. 1% ethanol) used for the GPC analyses was purchased from PENTA Ltd Czech Republic. d-Chloroform (CDCl_3_, 99.8%) for the NMR analysis was purchased from Sigma-Aldrich.

### 2.2 Transesterification of the castor oil

**Fig. 3:**
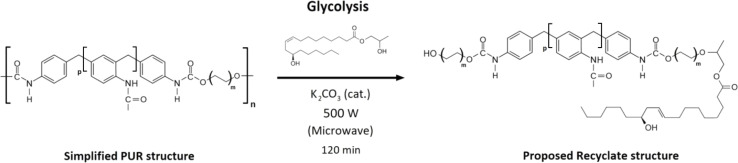
The reaction scheme of the commercial foam E 3943/134/LD glycolysis using 2-hydroxypropyl ricinoleate as a reactive solvent.

Castor oil (defined *M*_*n*_ of 926 g mol^−1^) was transferred into a round bottom flask (308 g, 0.33 mol), and propylene glycol (152 g, 2 mol) was added to the mixture. The catalyst (K_2_CO_3_) was mixed with the reactive components (9.2 g, 2 w%). The solution was set into the heating nest, and the reaction underwent a reflux of the solvent propylene glycol (temperature 188 °C). The transesterification process took 180 minutes. The termination time was determined *via* GPC analysis (the mixture contained only the product 2-hydroxypropyl ricinoleate). After the reaction, the excess propylene glycol and the formed glycerol were distilled from the mixture. The yield of the transesterification was determined gravimetrically (reached 97.5%). Most of the product was 2-hydroxypropyl ricinoleate (2-HPR) (as GPC and NMR analyses confirmed). The catalyst was left in the mixture since glycolysis was performed in the presence of the same catalyst. Therefore, this process is suitable for potential up-scaling purposes (Fig. 3).

^1^H NMR of 2-hydroxypropyl ricinoleate (500 MHz, chloroform-d) *δ*: 6.23 (d, *J* = 5.6 Hz, 1H), 5.56–5.41 (m, 2H), 4.15–4.05 (m, 2H), 3.98–3.87 (m, 1H), 3.76 (tdd, *J* = 6.8, 5.5, 1.7 Hz, 1H), 3.69 (d, *J* = 5.7 Hz, 1H), 2.32 (t, *J* = 8.5 Hz, 2H), 2.21–2.07 (m, 2H), 1.98–1.90 (m, 2H), 1.60 (ddd, *J* = 16.0, 8.6, 7.4 Hz, 2H), 1.50–1.08 (m, 21H), 0.93–0.85 (m, 3H).

ESI-MS of 2-hydroxypropyl ricinoleate analyzed precursor [M + K]^+^ 395.5 *m*/*z*. Fragments: 321.2 and 282.1 *m*/*z* according to.^41^

FTIR spectrum absorption wavenumber intervals: O–H stretch. 3550–3200 cm^−1^, C–H stretch. 3000–2840 cm^−1^, C=O (ester) stretch. 1750–1735 cm^−1^, C=C stretch. 1662–1626 cm^−1^, C–O (ester) stretch. 1210–1163 cm^−1^, C=C bend. 840–790 cm^−1^.

### 2.3 Glycolysis of the commercial foam with 2-hydroxypropyl ricinoleate

**Fig. 4:**
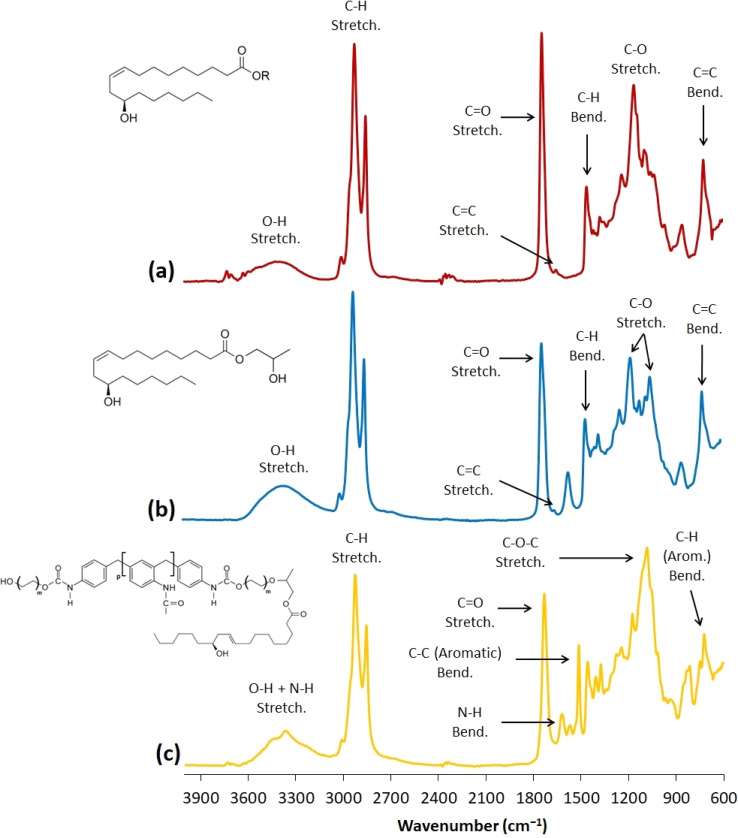
The FTIR spectra of castor oil (a), 2-hydroxypropyl ricinoleate (b), and the PUR liquid recyclate (c).

The produced 2-hydroxypropyl ricinoleate was used as the reactive solvent of the polyurethane foam glycolysis. The shredded PUR foam (255 g) was transferred into the microwave reactor with the 2-hydroxypropyl ricinoleate (382 g of 2-HPR containing 9.2 g of the catalyst K_2_CO_3_). The reaction mass ratio was 1 : 1.5 (foam : 2-HPR). The mixture was transferred into a 1 L round bottom flask with a PTFE shaft stirrer, set at a power of 500 W, and the reaction lasted 120 minutes. During the reaction, the foaming occurred, so the shaft stirrer rotation rate varied between 100–250 rpm to minimize the foaming. The generation of foam stopped after approximately 100 minutes. The eventually produced mixture (recyclate – REC) liquid yield was 87.2% (the commercial foam contains certain pigments, additives, and colorants, and their quantity in the foam is confidential). The yield was quantified gravimetrically (norm ASTM D5368-13). The catalyst was left in the product since it does not affect the addition of polyurethane forming reaction. The structural analysis of the produced recyclate was provided *via* FTIR analysis due to the complexity of the REC chemical structure (the original foam contains numerous components). The proposed simplified chemical structure based on the FTIR analysis is shown in Fig. 4. The volumetric and rheological characterization of REC was evaluated.

### 2.4 Reactivity investigation (cup tests) and the up-scaled foam production

The polyurethane formation reactivity, including the prepared recyclate (REC), was investigated *via* cup-test experiments. The polyol mixture containing REC (13 g) was poured into a 565 mL normalized cup (REC contents were 5%, 10%, 15%, 20%, 25%, and 30%). The percentages refer to the polyol part. Poly (methylene diphenyl diisocyanate) (PMDI) system Iso 123/6 (produced by BASF Ltd) was added into the polyol (23.4 g). The mixing ratio of polyol : isocyanate was 100 : 180 w/w. As soon as PMDI was transferred into the cup, the mixture was vigorously mixed at 1850 rpm for 10 seconds. The foaming reaction was monitored using ultrasound instrumentation, Ultraschall-Sensor P 8000 B, with the software Foam-Qualifying-System SQS-01 (BASF Ltd Certified). The values of cream time (beginning of the reaction) and rise time (end of the reaction) were extracted. Also, the block foam density was determined by the standardized gravimetric method (DIN EN ISO 845). The changes in the REC-containing polyol amine value were measured due to the rising reactivity of the system.

The up-scaling of the recyclate-containing foam was performed with the chosen REC content based on the cup test results. The increased polyol dosage containing 20% REC (1429 g) was mixed with Iso 123/6 PMDI system (2429 g) for 20 seconds at 1850 rpm. The mixing time is generally increased in the up-scaled quantities due to the complete homogenization. The mixed solution was poured into the 0.5 × 0.5 × 0.5 m mold (125 L), and the foaming reaction underwent. The process lasted 20 minutes to ensure the complete cure of the foam. After 24 hours, the prepared PUR block was cut into slices for the mechanical analyses.

### 2.5 Characterization methods

#### 2.5.1 Structural characterizations of the products

Gel permeation chromatography (GPC) determined the transesterification product molecular weight. Also, this method verifies the end of the reaction. The measurements were obtained *via* GPC (Agilent 1100, Santa Clara, CA, USA) in chloroform (CHCl_3_). The weight of the measured sample was 4 mg mixed in 1 mL of chloroform. Also, the mixture was filtered through a 0.22 μm PTFE filter to remove any solid particles. The analysis parameters were as follows: mobile phase flow 1 mL min; column temperature 30 °C, used column: PLgel 5 μm MIXED-C (300 × 7.5 mm). GPC was applied to the original castor oil and the produced 2-hydroxypropyl ricinoleate.

Nuclear magnetic resonance (NMR) was a structural verification method for the synthesized 2-hydroxypropyl ricinoleate. The instrument Bruker Avance III 500 MHz (Bruker, Billerica, MA, USA) was used for the experiments with the measuring frequency of 500 MHz for ^1^H NMR at the temperature of 30 °C using d-chloroform (CDCl_3_) as a solvent with tetramethylsilane (TMS) as an internal standard. The chemical shifts (*δ*) are shown in part per million (ppm) units. The resulting spectrum of 2-HPR is referenced by the solvent (d-chloroform). The coupling constant *J* has (Hz) unit with coupling expressed as s—singlet, d—doublet, t—triplet, q—quartet, p—quintet, m—multiplet.

Electrospray ionization mass spectrometry (ESI-MS) provided structural verification of produced molecules. The instrument used was Bruker EVOQ LC-TQ, which used electrospray ionization (ESI). Product scan spectra were obtained by fragmentation of the precursor ions. Collision energy spread (5–20 eV) enhanced the collected MS/MS data quality. Furthermore, the obtained mass spectra correspond with their prediction by CFM-ID 4.0,^41^ which also proposed the product ion structure for the most intensive masses.

Fourier-transform infrared spectroscopy (FTIR) contributed to the crossing structural analyses of the produced substances. FTIR served for the verification of the 2-HPR structure, the confirmation of the successful glycolysis of the PUR foam, the determination of the structural differences of the recyclate-containing PUR forming polyols, and FTIR evaluated the comparison of the virgin commercial foam structure and the recyclate-containing manufactured foam. The results were provided by the infrared spectrometer Bruker Tensor 27 (Billerica, MA, USA) by the attenuated total reflectance (ATR) method. A diamond was a dispersion component, and a diode laser was the irradiation source. The Michelson interferometer was used for the signal quantification. The spectra were composed of 32 scans with a measurement resolution of 2 cm^−1^.

The applied volumetric methods (hydroxyl number, amine number, acid number, and the water content) were determined using the particular norms. Hydroxyl number was measured following DIN EN ISO 4692-2. The acid number was determined using DIN EN ISO 2114. The amine value summarized for all prepared systems was detected *via* ISO 25761:2014. The water content was measured following ISO 14897:2023.

#### 2.5.2 Rheological characterization

The rheology (Brookfield RVDV-II + PX rotational viscometer) was studied to describe the apparent viscosity (*η*_app_) dependency on temperature due to its significant importance in the industry. The Arrhenius equation served for the dependency interpretation. Individual systems measuring volume was 500 μl. The measurements employed a Peltier platform with temperature regulation and the cone-plate geometry (40 mm, 2° angle). The measurements were performed at a shear rate of 100 s^−1^ and a temperature gradient of 25–60 °C. The measurement temperature step change was one measurement per every 1 °C.

The Arrhenius plot served for the summarization and calculation of rheological parameters.^42^ The equation defines the connection of the apparent viscosity changes with increasing temperatures as follows:1
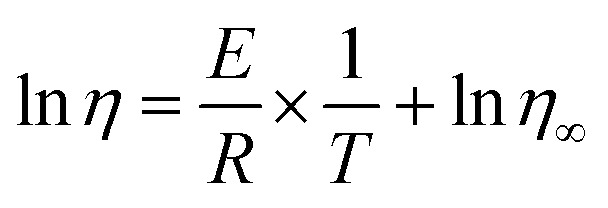
where *η* refers to the apparent viscosity (Pa s); *η*_∞_ stands for the pre-exponential factor (Pa s); *E* represents the flow activation energy (J mol^−1^); *R* is the universal gas constant (*J* (mol^−1^ K^−1^)) and *T* is the thermodynamic temperature (K).

#### 2.5.3 Tensile and flexural measurements of prepared foams

The particular norm (reference number ISO 1926:2005(E)) defined the foam tensile characterization. The elongation and the tensile strength were obtained from measurements. The flexural properties were determined according to ISO 1209-1:2007(E). The flexural strength and the flexural strain were obtained. The mechanical characterization was investigated for the standard E 3943/134/LD foam produced by BASF Ltd compared to the 20% REC-containing standard system. This strategy was selected based on the performed cup tests due to the reactivity levels of the 20% REC-containing system.

The norm ISO 1926:2005(E) defines the tensile strength (*σ*_MAX_) as follows:2
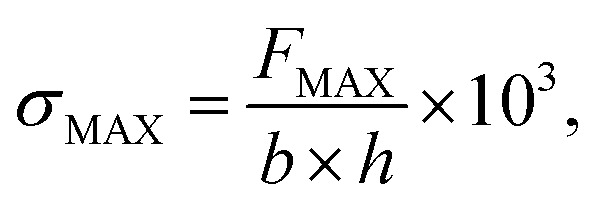
where *F*_MAX_ is the maximum force applied to the test subject (N), *b* is the width of the narrow parallel-sided section of the test subject (mm), and *h* is the thickness of the narrow parallel-sided section of the test subject (mm). The following equation defines elongation (*e*):3
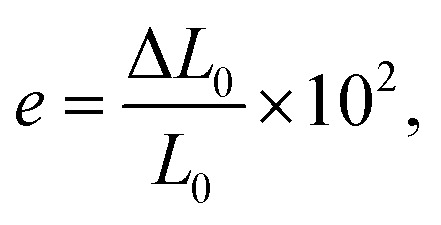
where *L*_0_ is the test subject's original length (mm), and Δ*L*_0_ is the increased length of the test subject at a given force interval (mm).

The norm ISO 1209-1:2007(E) describes the flexural measurements. The equation defining the flexural strength (*σ*_FLEX_) is formulated as follows:^43^4
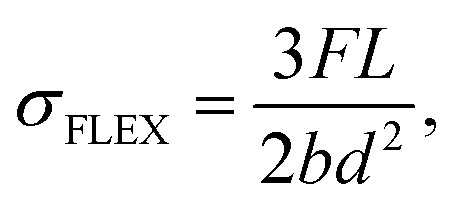
where *F* is the force at 24% of strain (N), *L* is the test subject length (mm), *b* is the test subject width (mm), and *d* is the test subject thickness (mm).

## 3 Results and discussion

### 3.1 Synthesis of 2-hydroxypropyl ricinoleate and PUR glycolysis

The transesterification of the castor oil, resulting in 2-hydroxypropyl ricinoleate (2-HPR) formation, was performed to obtain a polyol with sufficient chain-extending characteristics. Since the hydroxyl group in ricinoleic acid is localized on the ω-7 carbon, most of the acid carbon backbone serves as a chain extender in polyol-containing structures. Additionally, the 2-hydroxypropyl ester of this fatty acid is not used in material chemistry and has an innovative and functional potential as a glycolysis agent for PUR recycling. Propylene glycol can be obtained in a bio-based quality, and castor oil is also a renewable source. The base-catalyzed transesterification includes the advantage of the catalyst transfer to the following reaction since glycolysis can also undergo in the presence of a base. Thus, the suggested synthesis process can be efficiently up-scaled and transferred in the manufacture. The synthesized 2-HPR was structurally verified *via* crossed analysis (GPC, FTIR, ^1^H NMR). The obtained FTIR spectra of castor oil, 2-hydroxypropyl ricinoleate, and the PUR liquid recyclate are shown in Fig. 4(a)–(c). The gravimetrical yield was 97.5% (previously mentioned in the Experimental section). The following glycolysis in a microwave reactor incorporating previously synthesized 2-HPR with the catalyst (K_2_CO_3_) resulted in a gravimetrical yield of 87.2% due to the unreacted commercial foam additives occurring as solid particles in the reaction mixture. The volumetric analysis results are summarized in Table 1. The PUR liquid recyclate contained 1.1 wt% of water. Water is an essential component for PUR formation since it causes the blowing reaction during the foaming. The studied system E 3943/134/LD contains a certain amount of water, and the water content in the PUR liquid recyclate was increased to the content present in the original virgin polyol system. The exact water amount cannot be mentioned due to the trading secret. The glycolysis temperature monitoring is summarized in Table 2. Also, complex volumetric analyses were performed with REC since they confirmed the presence of functional groups (amine, hydroxyl, or acidic).

**Table 1:** The measured volumetric parameters of PUR recyclate (REC)

The PUR liquid recyclate (REC)		
Parameter	Unit	Value
Hydroxyl value	mg KOH per g	280.5
Amine value	mg KOH per g	74.95
Acid value	mg KOH per g	0.5
Water content	w%	1.1

**Table 2:** The temperature changes during the glycolysis process

Glycolysis temperature	
Reaction time (min)	Temperature (°C)
0	23.4
10	64.8
20	113.4
30	146.7
40	149.5
60	151.5
80	150.4
100	150.6
120	149.8

The obtained FTIR results confirmed the structural changes of castor oil, resulting in the 2-HPR formation. Although 2-HPR structural verification was provided *via*^1^H NMR and ESI-MS, FTIR detected the signal increase in the O–H stretching area of the 2-HPR spectrum which verifies the hydroxyl functional group content rise. This is caused by the additional O–H group in the propylene glycol structure. Also, the C=O stretching signal in the 2-HPR spectrum changed. The intensity decreased since the triacylglyceride structure was modified *via* the transesterification. This modification caused the C=O signal to decline due to the ester bonding reduction. Additionally, the spectrum (c) in Fig. 4 provided the structural characterization of the glycolyzed PUR. Mainly, the amine groups occurred in the recycled PUR liquid (3600–3200 cm^−1^ and 1620–1590 cm^−1^) with the aromatic structures (1520–1490 cm^−1^ and 790–750 cm^−1^). This structural characterization, which is connected to the volumetric analysis results, confirms the transesterification and glycolysis.

The GPC results uncover a negligible amount of other products (esters of other present carboxylic acids), which contribute to the number-average molecular weight of synthesized 2-HPR. However, the retention time shift combined with the analyzed molecular weight confirms the presence of 2-HPR in most of the yield (98.7% after the calculation). Also, the obtained ^1^H NMR spectrum with integrated and analyzed signals verifies the presence of a 2-HPR structure. The confirmation was provided by the obtained FTIR spectrum of the 2-HPR product, where the essential functional groups can be found. The REC FTIR spectrum (Fig. 4(c)) confirms the structural changes involving mainly amine functional groups, the increase of the hydroxyl group signals, and the occurrence of the aromatic bonds in the spectrum. All these verifications prove the incorporation of the urethane bonds and the presence of aromatic structures in the product (REC).

### 3.2 The reactivity and properties of the REC-containing PUR systems

**Fig. 5:**
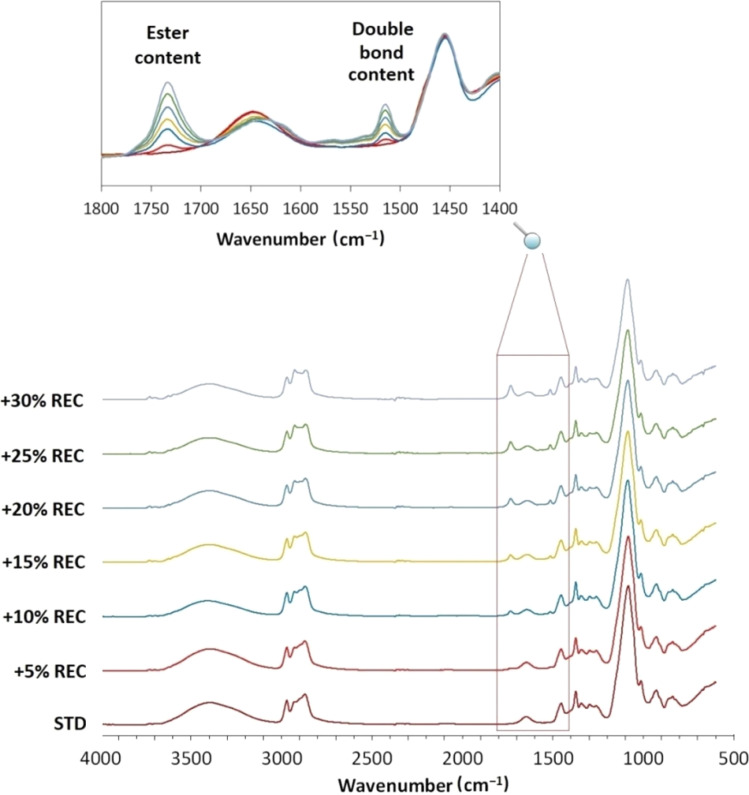
The structural changes of REC-containing polyol systems monitored by FTIR with emphasized wavenumber interval regarding an ester and a double bond presence.

The polyol systems with particular isocyanate were mixed with the prepared PUR recyclate, forming an increasing REC-containing series (from 5 w% to 30 w% REC). The prepared systems were volumetrically analyzed due to the considerable amine number. Amine functional groups catalyze the blowing reaction and increase the foaming rate.^44^ Also, the mixtures' rheological characterization was investigated to sufficiently describe the flow properties of REC-containing polyols, which are critical factors for industrial manufacturing. Moreover, the structural changes were scrutinized *via* FTIR, especially the increasing presence of an ester in polyol since BASF Ltd certified polyol consists mainly of polyethers. The summarized complete composition of the prepared REC-containing polyol mixtures with incorporated polyol : iso mixing ratio is shown in Table 3 together with the calculated rheological parameters. The graphical interpretation of the systems' rheology is present in ESI (Fig. S4†). The FTIR spectrum comparison is displayed in Fig. 5.

**Table 3:** The summarized composition and rheological properties of studied REC-containing polyol systems for the PUR preparation

Composition				Rheology				
	Recyclate content (%)	Polyol : PMDI ratio (w/w)	Amine value (mg KOH per g)		*η*_30°C_ (mPa s)	*η*_∞_ (mPa s)	*E* (kJ mol^−1^)	*R* ^2^
STD	0	100 : 180	2.3	STD	274	8.3 × 10^−7^	32.1	0.99
+5%	5		5.9	+10%	187	5.8 × 10^−8^	37.8	0.99
+10%	10		9.6					
+15%	15		12.9	+20%	129	2.8 × 10^−8^	38.6	0.99
+20%	20		16.8					
+25%	25		19.7	+30%	109	1.2 × 10^−8^	40.4	0.98
+30%	30		24.1					

The rheological characterization uncovered the apparent viscosity decrease with the rising REC content. The viscosity value at 30 °C for a 30% REC system resulted in a 60.2% decrease. This outcome is significantly beneficial for the manufacturing process since fewer requirements are applied for the dosage systems and the transfer pumps. Also, industrial mold filling is more convenient if the system exhibits lower viscosity. The complete rheological characterization up to 60 °C was performed due to the additional processes connected to the industrial production. The PUR-forming systems are transported from the producing facility to the destination where the PUR products are manufactured. The flow characteristics vary during the expedition transfers, where the liquid systems exhibit different viscosity levels. Generally, the lower the viscosity at the increased temperature, the better for the industrial purposes since the transfer is faster and more convenient. The results uncovered the decreasing viscosity levels at higher temperatures, similar to the most important working temperature at approximately 30 °C.

Fig. 5 uncovers the structural changes that have occurred with the increase in PUR liquid recyclate content. The marked and detailed signals referring to ester C=O bonding and unsaturated C=C bonds especially change with the rising REC content. This analysis verifies the ester-origin content in the formed PUR, which is essential information regarding the potential multiple chemical recycling. The increase in the ester content in the PUR might affect the product's faster hydrolysis. Also, it could serve as a detecting tool for the chemically recycled PUR obtained from the different polyurethane producers. The increase of C=C bonding additionally verifies the rising content of unsaturated carbon chains in the oil structure.

**Fig. 6:**
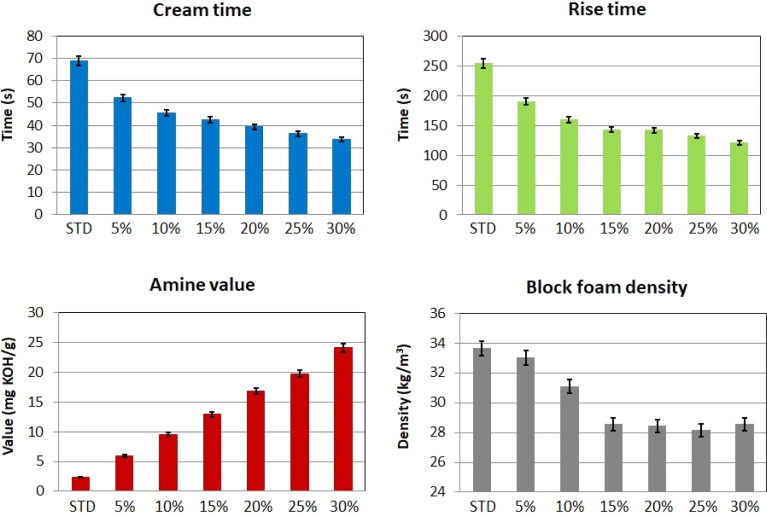
The REC-containing polyol systems cup test results and the amine value and density changes of the studied mixtures.

The increasing systems' amine number tremendously affected the reactivity of the polyols during the foaming reaction. This phenomenon was observed in previous publications.^45^ The cup test results are shown in Fig. 6. The cream time and the rise time decreased by 51.2% and 52.8%, respectively, with 30% of REC content in the mixture. Reducing blowing catalyst content in the virgin standard mixture could solve these reactivity changes. The deamination is an alternative;^46^ this process complicates the manufacture and involves more expenses. According to the manufacturing process, the cream time above 40 seconds is acceptable for industrial applications. Therefore, the system containing 20% REC (the cream time of 39.4 seconds) was chosen to be up-scaled and further mechanically studied. This system involves a considerable amount of recycled content and can be used on an industrial scale. Also, the block foam density decreased by 14.8% with the 30% of REC. Also, the density reduction reaches an approximately constant value at 15% REC content. This result is positive since the industry tends to limit the vehicle's weight. This factor can be positive if the mechanical properties of the REC-containing foams do not worsen. The mechanical properties study is performed in the following section. The increase in reactivity might also be influenced by the remaining K_2_CO_3_ catalyst used for oil transesterification and PUR glycolysis.^47^ Although particularly potassium acetate was mentioned in relation to the reaction with isocyanate systems, potassium carbonate might have affected the PUR-forming process.

### 3.3 The up-scaling of REC-containing foams

**Fig. 7:**
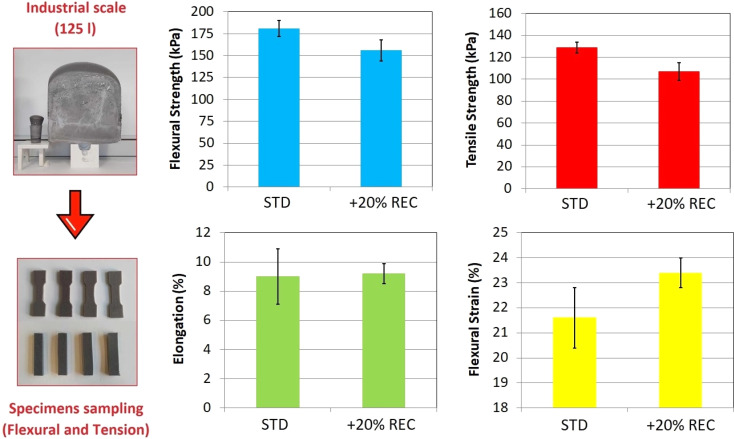
The up-scaling of the 20% REC-containing PUR foam and the tensile and flexural results comparison of standard and recyclate-containing system.

The REC-containing foam up-scale in 125 L volume is a critical experimental parameter. This scaling is industrially significant since manufacturing errors such as spontaneous ignition, foam collapsing, or a surface bubble formation can occur during the process. The prepared foam block containing 20% REC is displayed in Fig. 7 compared to the laboratory 565 mL cup. The block was formed without any mentioned complication. The chosen REC content (20%) also did not cause any problems during the experiment. However, the cream time of around 40 seconds was the least acceptable since the foam rise began exactly after the dosage was transferred (20 seconds of homogenization and 20 seconds of the mixture transfer). The faster foam rise damages the initially formed system surface and causes the central collapse of the foam.^48^ The mechanical analyses test specimens were cut off the foam slices (Fig. 7). The results of the tensile and flexural mechanical tests are also shown in Fig. 7.

The tensile properties study resulted in the REC-containing foam tensile strength decrease of 17.0% while the elongation absolute value remained statistically similar (the deviation of measured 6 samples was lower in REC-containing foam). The decrease in tensile strength is not industrially significant since PUR foam forms the core of the applied sandwich composite. On the other hand, tensile elongation plays a more important role in the application since the manufactured products are often flexed and bent. Therefore, the tensile results confirm the positive effect on the produced PUR foam since the elongation statistically tends to be more consistent in the case of 20% REC foam (based on the elongation deviations). The 20% REC foam flexural strength decreased by 13.8%. However, the flexural strength lowering signalizes the lower applied force on the foam. Therefore, the foam does not require to be bent forcefully. Additionally, the flexural strain of 20% REC foam increased by 8.3% while exhibiting a lower deviation than the standard foam (similar to the tensile elongation tests). Also, one standard foam specimen cracked during the flexural tests, and the overall standard foam test subject recorded less stabilized flexural strength values than 20% REC foam. Eventually, the foam containing 2-HPR and recycled PUR reached better industrial potential than the standardized E 3943/134/LD foam.

**Fig. 8:**
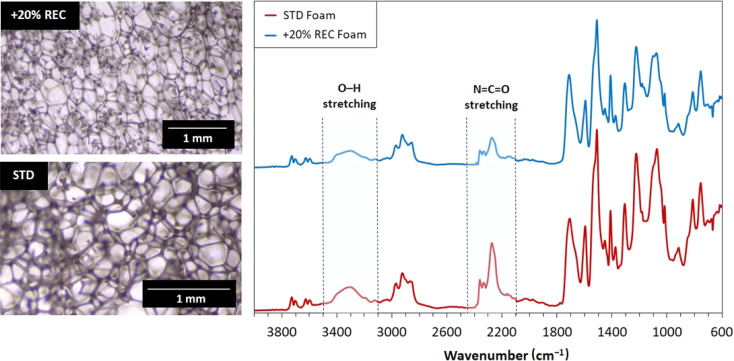
The optical microscopy images and FTIR analyses of standard (STD) and 20% REC-containing PUR foams.

The foam molecular structural changes were investigated *via* FTIR. The pore structure was monitored by optical microscopy. The results of performed analyses are shown in Fig. 8. The isocyanate and hydroxyl bond signals decreased in the case of 20% REC foam. This outcome probably signals the more quantitative reaction forming more urethane bonds. The pore structure images reveal that standard foam consists of smaller pores than the 20% REC foam. The pore walls form similar diameters in both systems. The hexagon-shaped cells typically appear in PUR foams, and this signature parameter is maintained in both systems.

## 4 Conclusions

This work evaluated a suggested polyurethane (PUR) recycling process involving 2-hydroxypropyl ricinoleate (2-HPR) as a reactive diluent produced from castor oil. The commercial PUR foam produced by BASF Ltd was depolymerized *via* glycolysis using 2-HPR and then incorporated into the PUR-forming mixture to produce recyclate-containing foam. The REC-containing foam was applied in automotive as a vehicle headliner composite core component. Firstly, 2-HPR synthesis was performed, resulting in a yield of 97.5%. The structural verification of formed 2-HPR was provided by GPC, FTIR, ESI-MS, and ^1^H NMR analyses. Consecutively, the PUR liquid recyclate (REC) was produced using 2-HPR as a glycolysis diluent. The REC yield reached 87.2% due to the insoluble additives of the commercial foam. The significant structural characterization (presence of functional groups) was provided *via* FTIR. Since the PUR liquid recyclate is a multi-component system, the structural verification focused on the functional groups (amine, hydroxyl, ester). The REC-containing PUR forming liquid polyol mixtures rheological study, FTIR characterization, and reactivity investigation were provided to select the most prospective REC content for the up-scaling. The REC content in the polyol mixture decreased the apparent viscosity (*η*_30°C_) levels (standard polyol *η*_30°C_ was 274 mPa s while 30% REC-containing polyol *η*_30°C_ reached 109 mPa s). The decrease in viscosity is a positive factor since it increases the efficiency of the manufacturing process. The FTIR analyses confirmed the increase in the ester amount and double bond number caused by the presence of 2-HPR. Standardized cup tests provided the reactivity investigation. The results uncovered the significant impact on the reactivity by the increased amine value of the system (amine values of standard foam and +30% REC foam reached 2.3 and 21.4 mg KOH per g, respectively). The block foam density reached a 14.8% decrease compared to the standard foam and +15% REC foam, and then it remained practically constant. The decreased block foam density is perceived as a positive factor since it ensures the product's weight reduction. Based on the laboratory test, the system containing +20% REC was chosen for the scale-up. The tensile test results uncovered the REC-containing foam tensile strength decrease (107 ± 8 kPa) compared to the standard foam (129 ± 5 kPa). At the same time, the elongation remained statistically constant, including a higher deviation in the case of standard foam (REC-containing elongation 9.2 ± 0.7% and standard elongation 9.0 ± 1.9%). The flexural tests uncovered the REC-containing foam flexural strength decrease (156 ± 12 kPa) and flexural strain deformation limit increase (23.4 ± 0.6%) in comparison with the standard foam flexural strength (181 ± 9 kPa) and flexural strain deformation limit (21.6 ± 1.2%). The results of the mechanical tests confirm that the presence of 20% PUR recyclate using 2-hydroxypropyl ricinoleate in standard foam enhances the flexural properties of applied composites. Therefore, the recyclate incorporated into the manufacturing process positively impacts the products.

## Data availability

The data supporting this article have been included as part of the ESI.†

## Conflicts of interest

The authors declare no competing financial interest.

## Supplementary Material

RA-014-D4RA04972A-s001
