# Supplementary material for: Chemically recycled commercial polyurethane (PUR) foam using 2-hydroxypropyl ricinoleate as a glycolysis reactant for flexibility-enhanced automotive applications
Source: RSC Adv. 2024 Sep 20;14(41):29966–78. doi: 10.1039/d4ra04972a (PMC11413739; doi:10.1039/d4ra04972a)
Supplement: RA-014-D4RA04972A-s001 [file RA-014-D4RA04972A-s001.pdf]

Supplementary Information (SI) for RSC Advances.
This journal is © The Royal Society of Chemistry 2024

Supporting Information

Chemically recycled commercial polyurethane (PUR) foam using 2-hydroxypropyl ricinoleate as a glycolysis reactant for flexibility-enhanced automotive applications

Vojtěch Jašek[a*], Petr Montag[b,c], Přemysl Menčík[a], Radek Přikryl[a], Alena Kalendová[b], Silvestr Figalla[a]

a Institute of Materials Chemistry, Faculty of Chemistry, Brno University of Technology, 61200 Brno, Czech Republic.

b Tomas Bata University in Zlin, Faculty of Technology, Department of Polymer Engineering, 76001 Zlín, Czech Republic

c BASF Ltd., Czech Republic

*corresponding author: xcjasekv@vutbr.cz

Table of content

1. **Structural analyses of the products**
   - **Figure S1.** The GPC analysis of castor oil and the synthesized 2-hydroxypropyl ricinoleate.
   - **Figure S2.** The ^{1}H NMR spectrum of the synthesized 2-hydroxypropyl ricinoleate.
   - **Figure S3.** The ESI-MS spectrum of the synthesized 2-hydroxypropyl ricinoleate.
2. **The graphical interpretation of recyclate (REC) containing polyols rheological study.**
   - **Figure S4.** The apparent viscosity dependency on temperature and the graphical interpretation of the Arrhenius equation.
3. **Cup test ultrasound measured reactivity results**
   - **Figure S5. – Figure S11.** The cup test results of the foam mixtures.
4. **The mechanical properties of standard E 3943/134/LD and +20% REC foams**
   - **Figure S12.** The results of foam tensile tests. (a) The tensile study of standard E 3943/134/LD foam, (b) The tensile study of +20% recyclate (REC) foam.
   - **Figure S13.** The results of foam flexural tests. (a) The flexural study of standard E 3943/134/LD foam, (b) The flexural study of +20% recyclate (REC) foam.
5. **The additional pictures of formed REC-containing PUR foams**
   - **Figure S14.** The formed PUR cups from the performed cup tests. The percentage on the cup marks the wt.% amount of the PUR liquid recyclate in the foam.
   - **Figure S15.** The quantity comparison of 565 ml cup PUR and 125 L PUR mini block containing 20 wt.% of the PUR liquid recyclate.

1. Structural analyses of the products

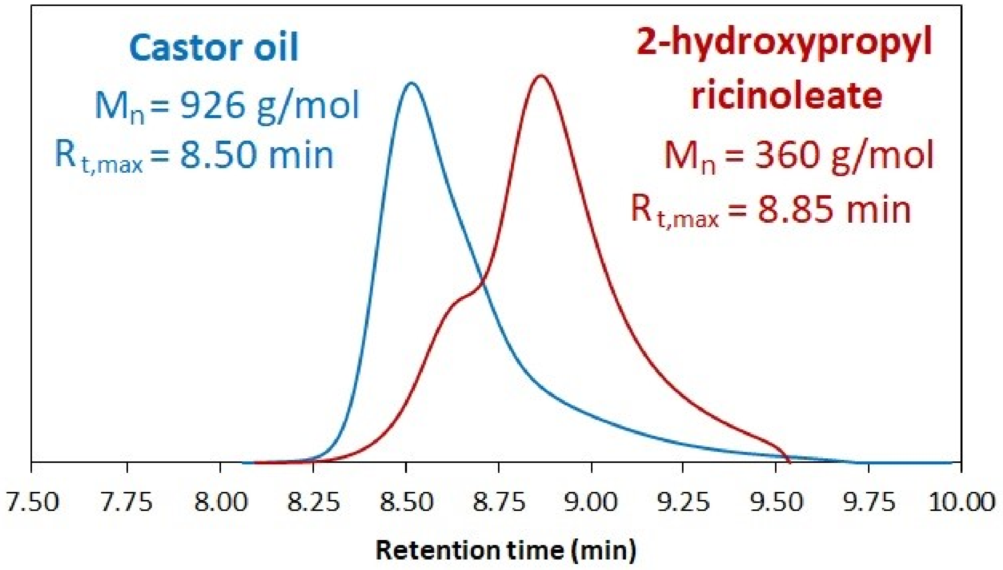

Figure S1. The GPC analysis of castor oil and the synthesized 2-hydroxypropyl ricinoleate.

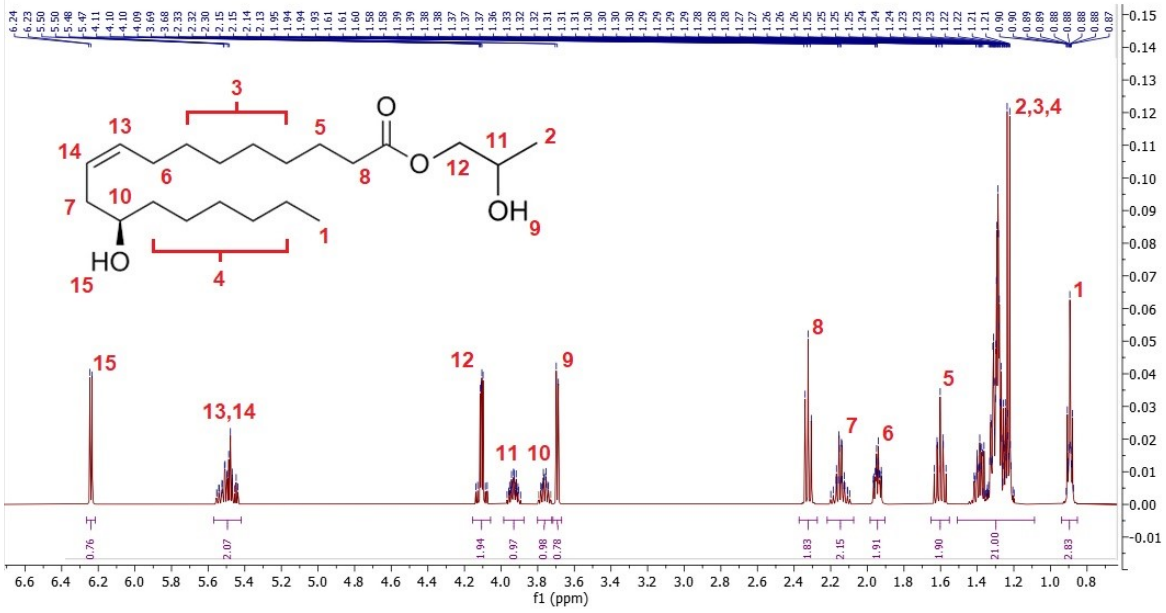

Figure S2. The ^{1}H NMR spectrum of the synthesized 2-hydroxypropyl ricinoleate.

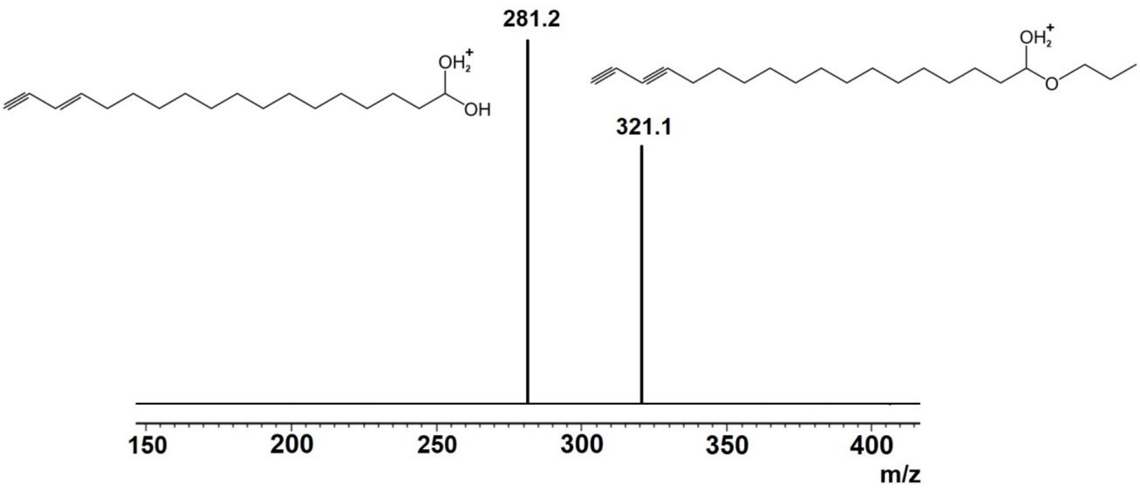

Figure S3. The ESI-MS spectrum of the synthesized 2-hydroxypropyl ricinoleate.

2. The graphical interpretation of recyclate (REC) containing polyols rheological study.

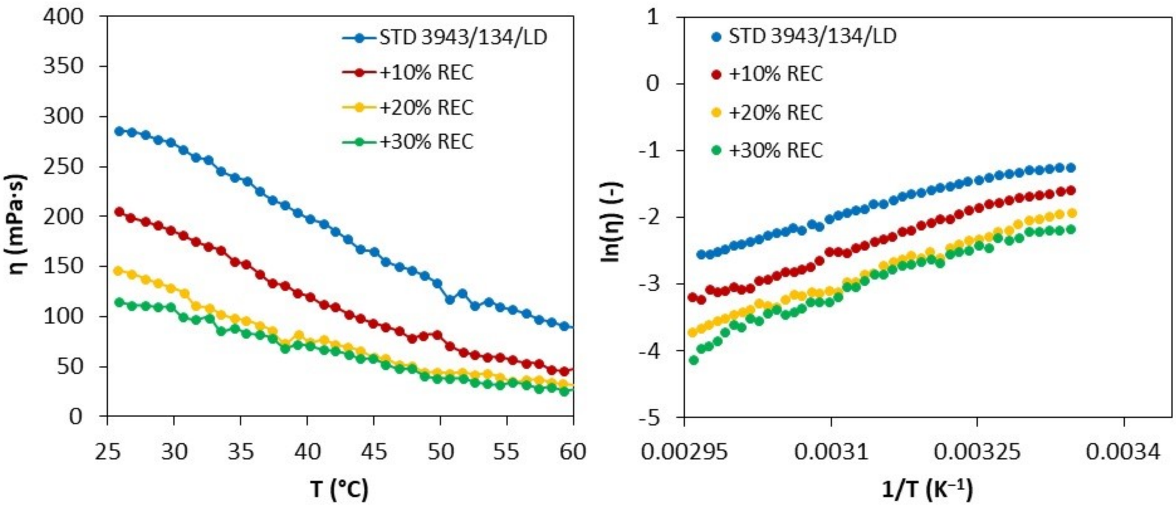

Figure S4. The apparent viscosity dependency on temperature and the graphical interpretation of the Arrhenius equation.

3. Cup test ultrasound measured reactivity results

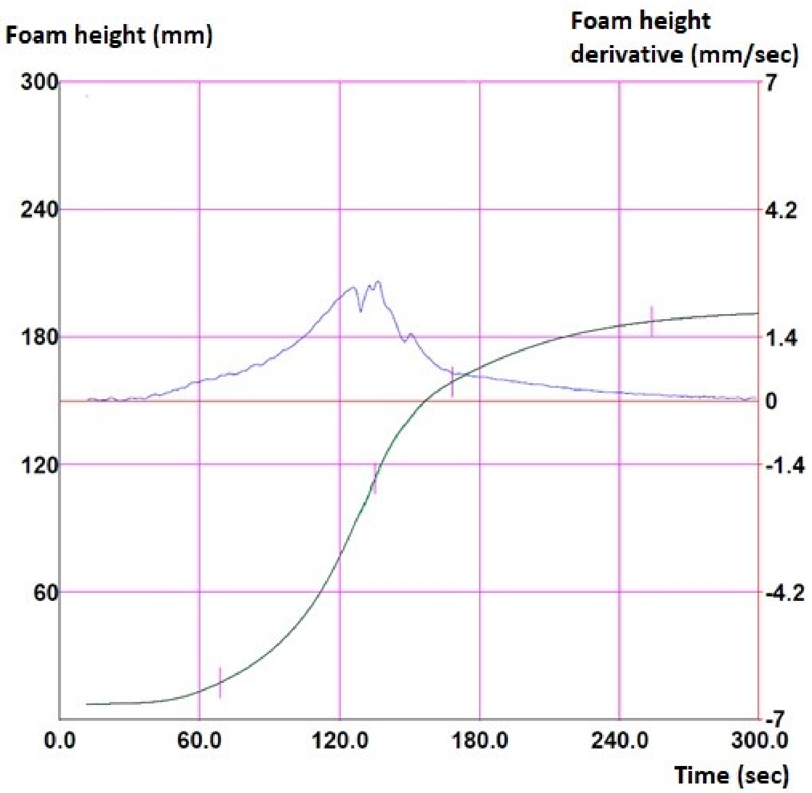

Figure S5. The cup test results of E 3943/134/LD standard polyol (STD).

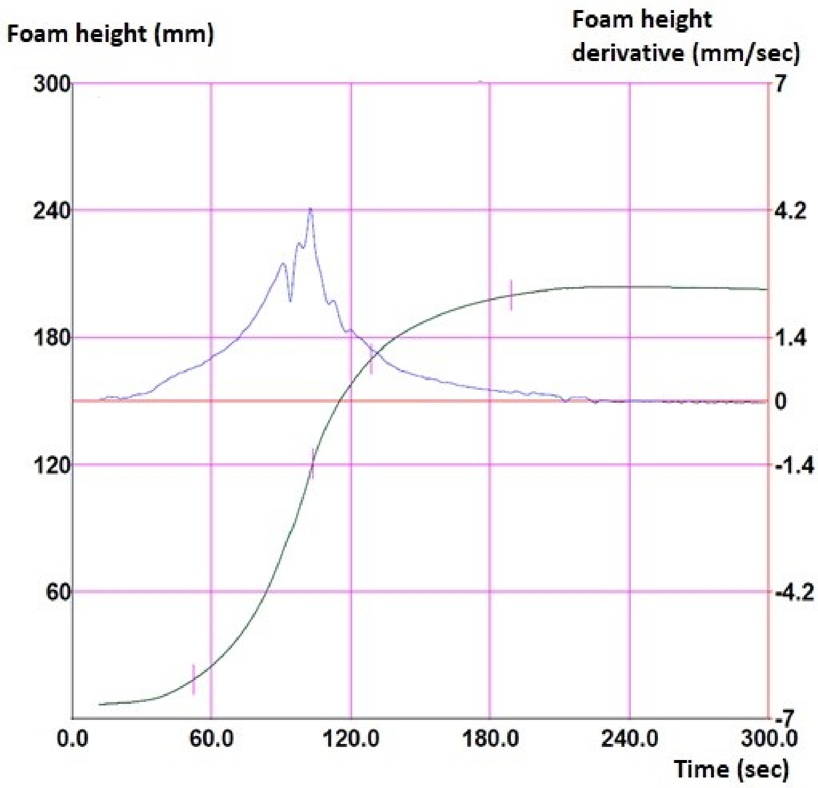

Figure S6. The cup test results of the mixture with added 5% PUR recyclate (REC).

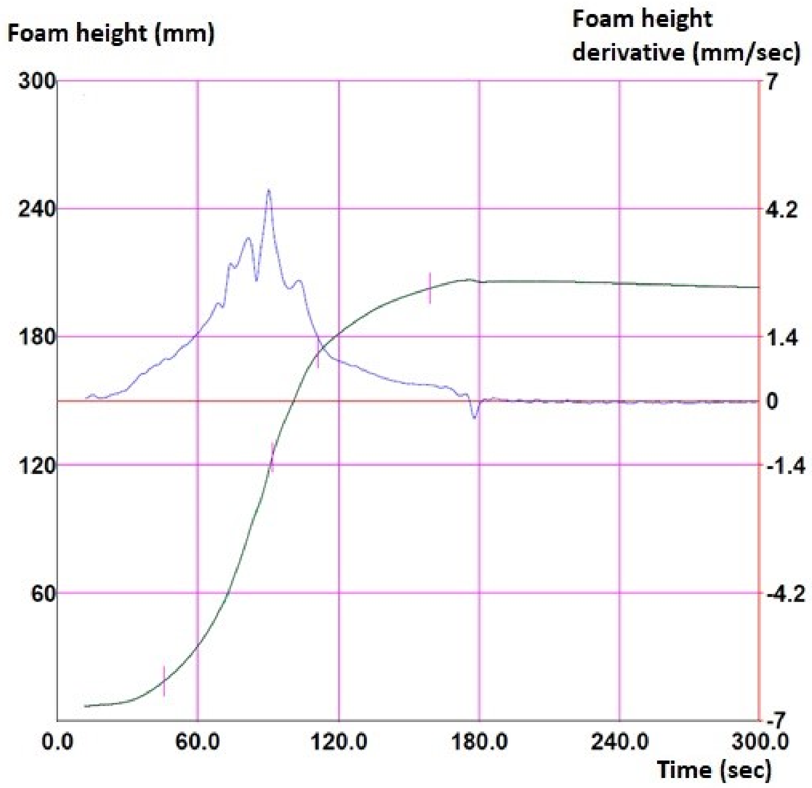

Figure S7. The cup test results of the mixture with added 10% PUR recyclate (REC).

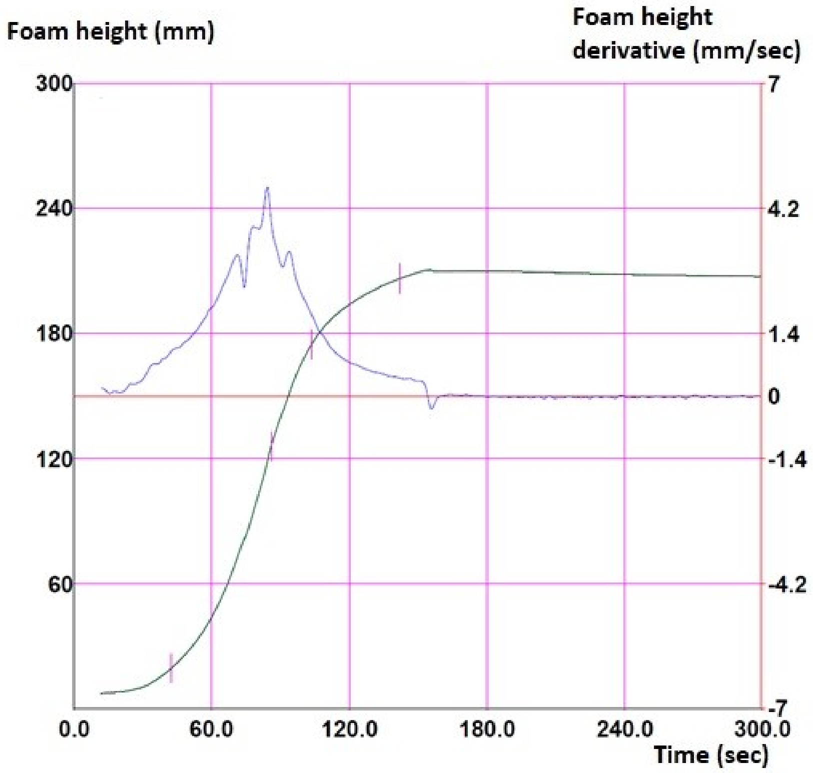

Figure S8. The cup test results of the mixture with added 15% PUR recyclate (REC).

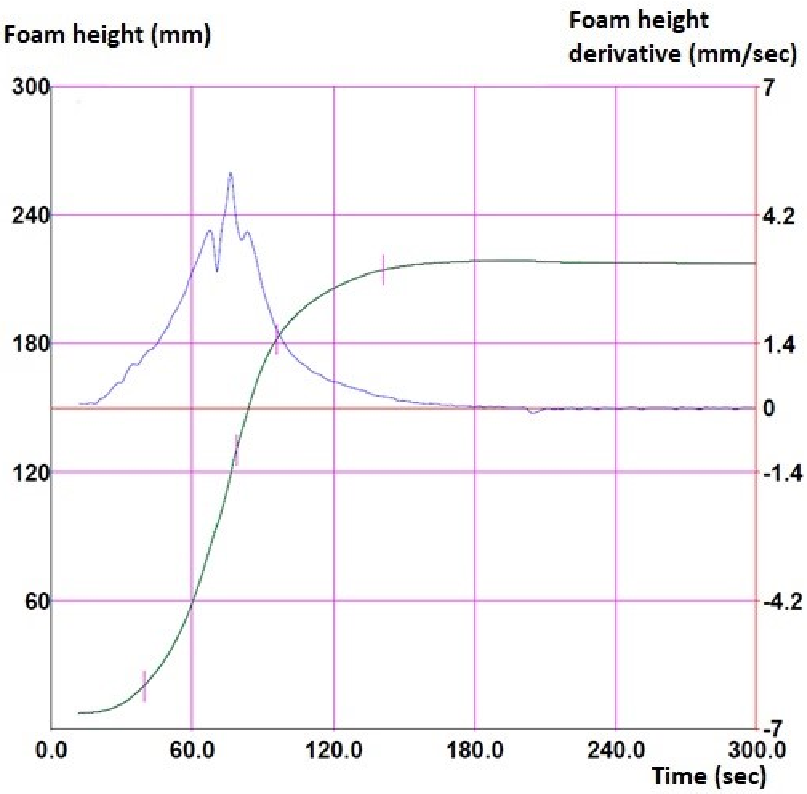

Figure S9. The cup test results of the mixture with added 20% PUR recyclate (REC).

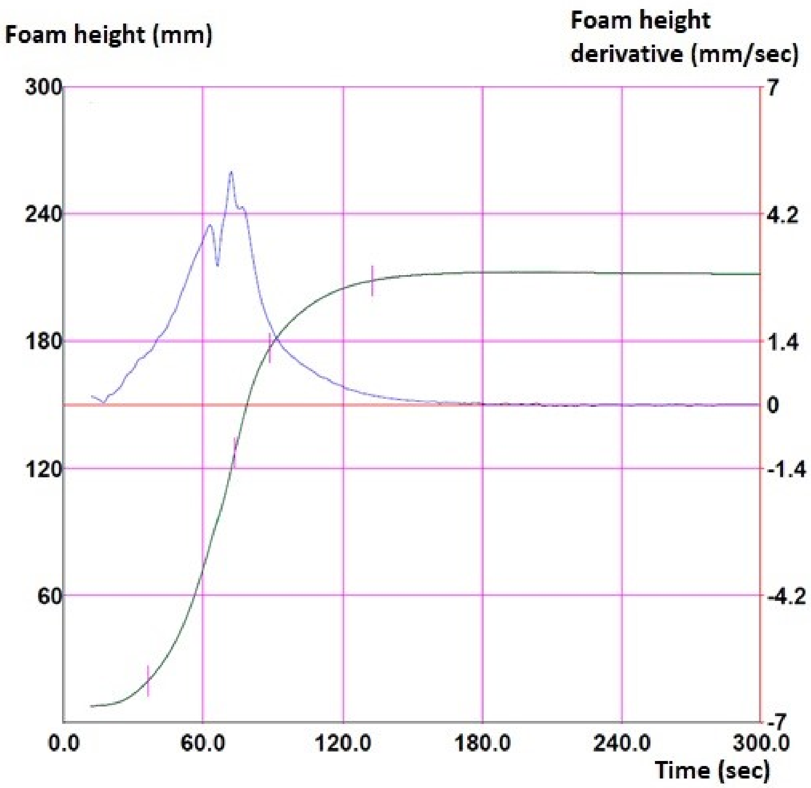

Figure S10. The cup test results of the mixture with added 25% PUR recyclate (REC).

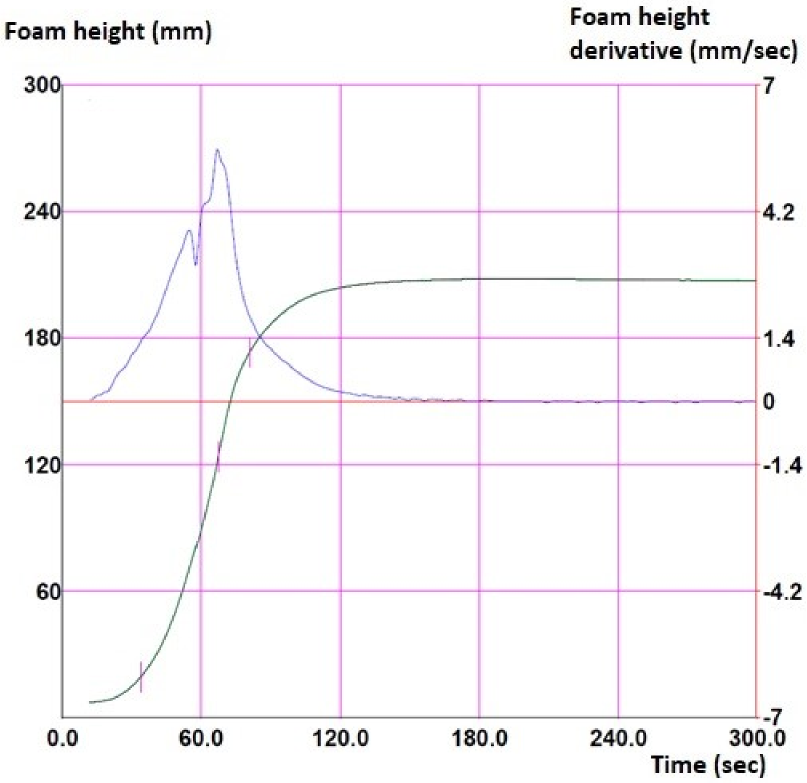

Figure S11. The cup test results of the mixture with added 30% PUR recyclate (REC).

4. The mechanical properties of standard E 3943/134/LD and +20% REC foams

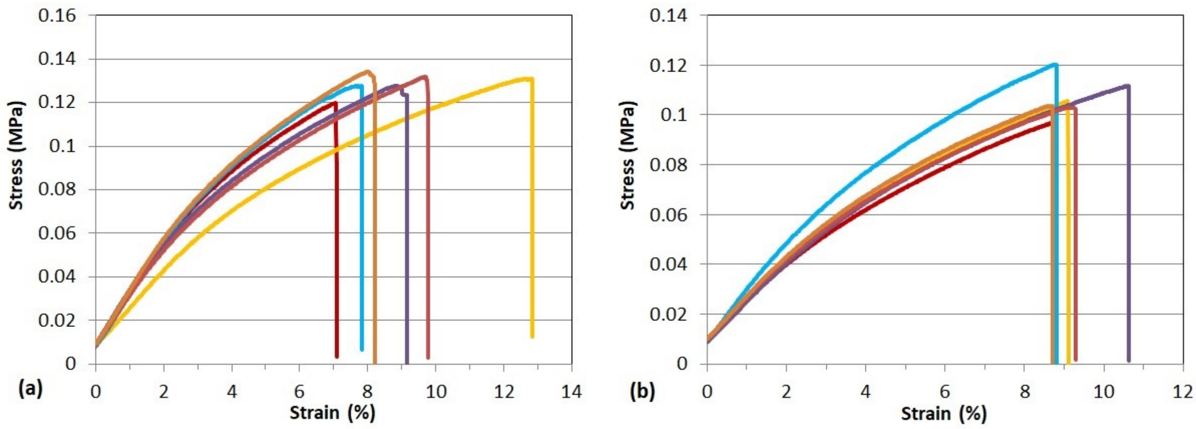

Figure S12. The results of foam tensile tests. (a) The tensile study of standard E 3943/134/LD foam, (b) The tensile study of +20% recyclate (REC) foam.

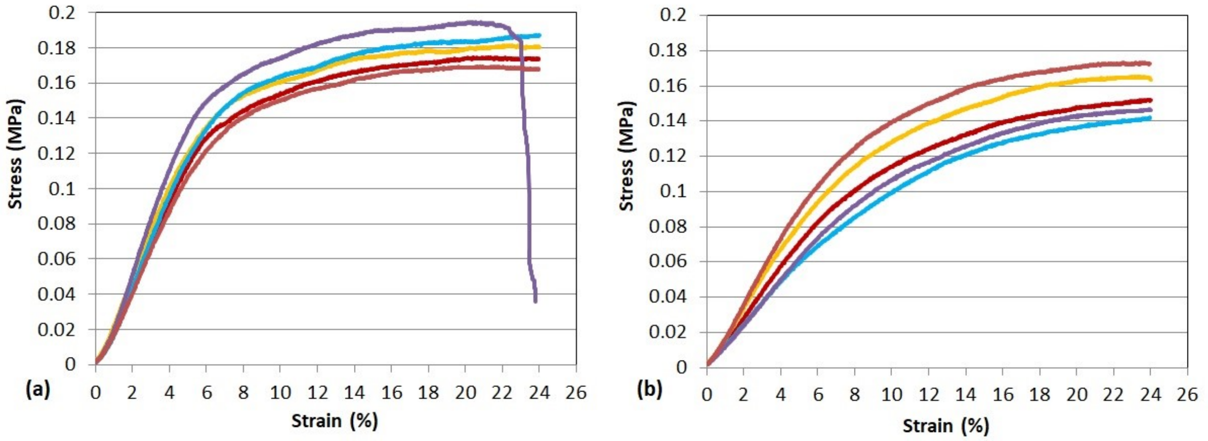

Figure S13. The results of foam flexural tests. (a) The flexural study of standard E 3943/134/LD foam, (b) The flexural study of +20% recyclate (REC) foam.

5. The additional pictures of formed REC-containing PUR foams

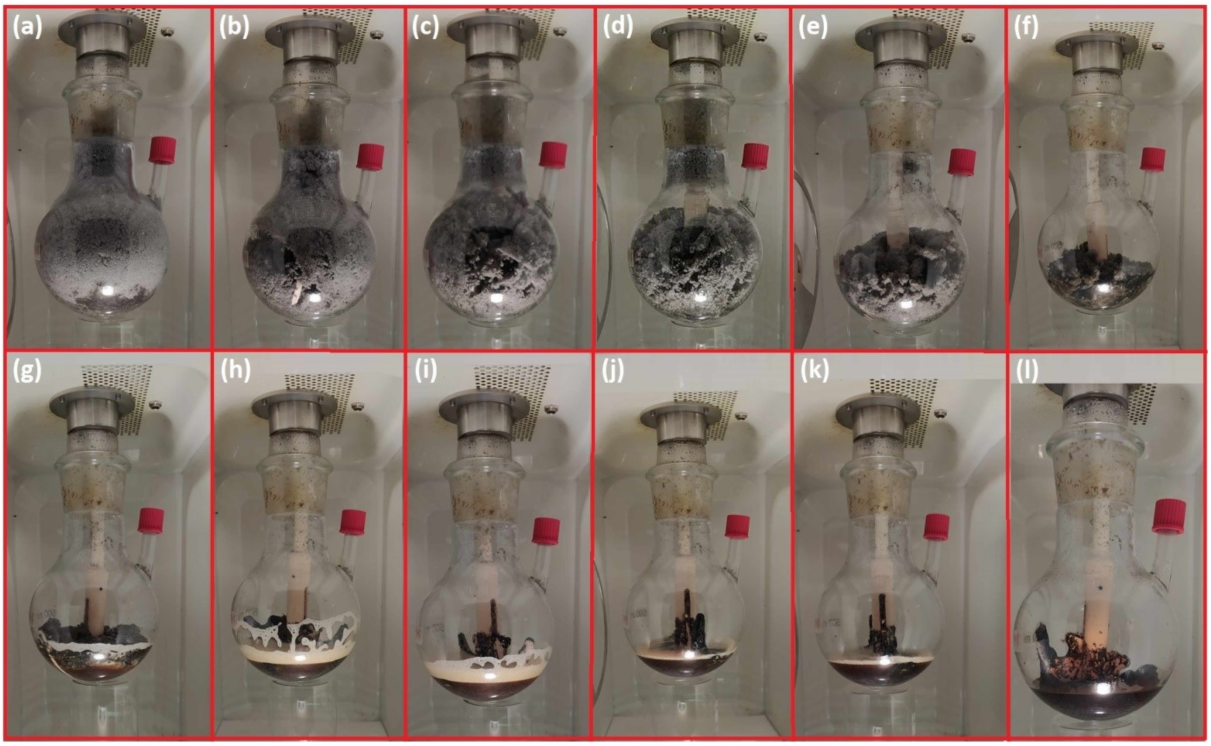

Figure S14. The glycolysis reaction progress in time. (a) 10 minutes, (b) 20 minutes, (c) 30 minutes, (d) 40 minutes, (e) 50 minutes, (f) 60 minutes, (g) 70 minutes, (h) 80 minutes, (i) 90 minutes, (j) 100 minutes, (k) 110 minutes, (l) 120 minutes.

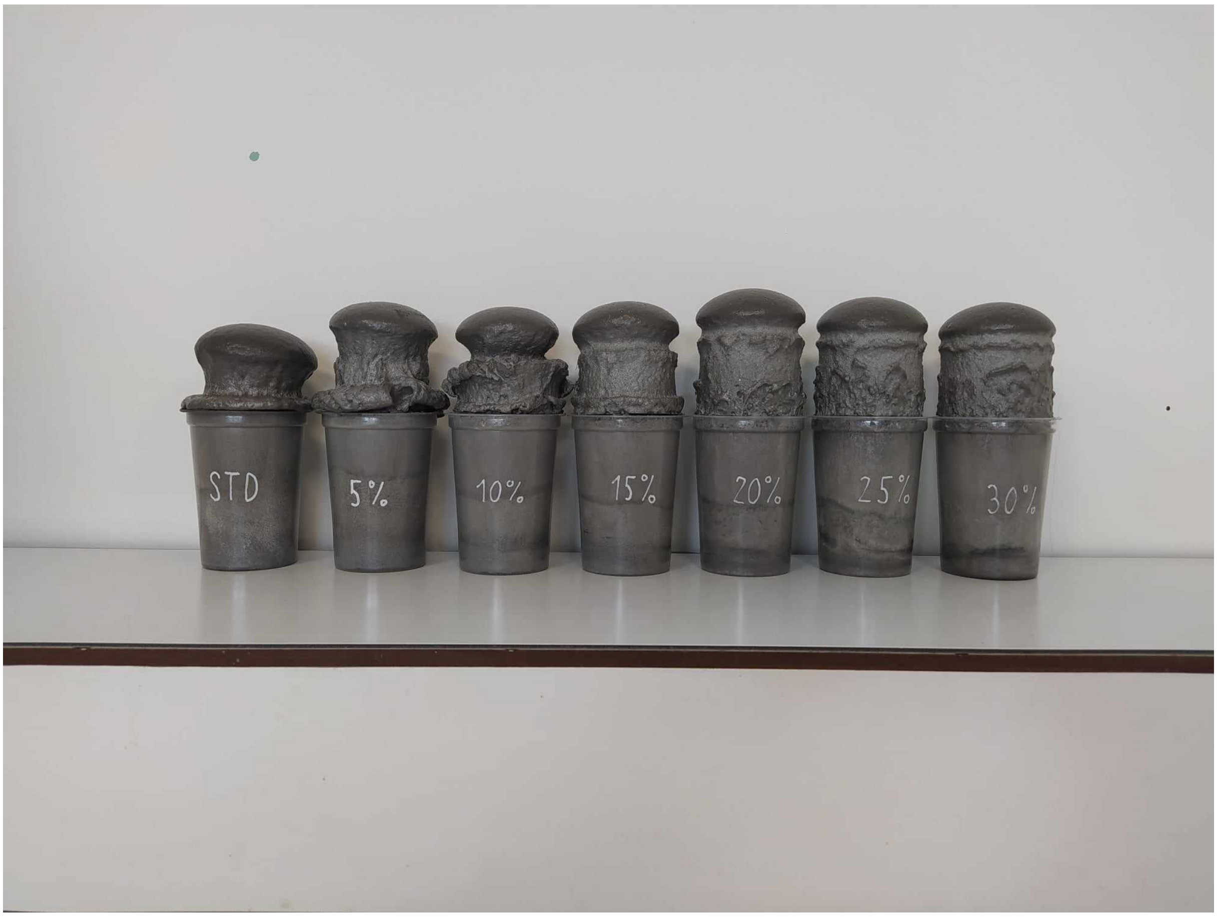

Figure S15. The formed PUR cups from the performed cup tests. The percentage on the cup marks the wt.% amount of the PUR liquid recyclate in the foam.

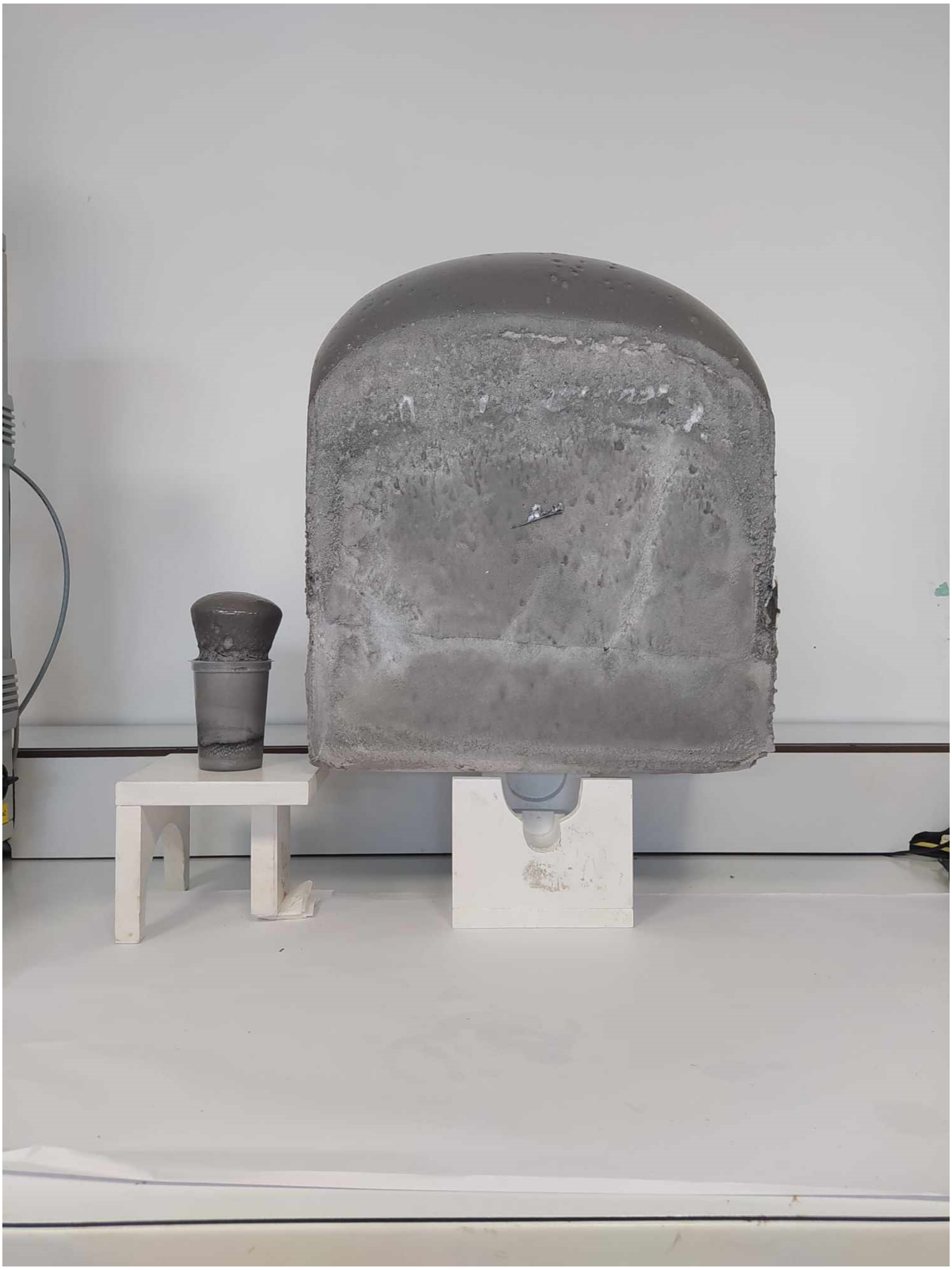

Figure S16. The quantity comparison of 565 ml cup PUR and 125 L PUR mini block containing 20 wt.% of the PUR liquid recyclate.
